# Clinical best practices for caring for people with expanded resistance to newer TB drugs

**DOI:** 10.5588/ijtldopen.25.0240

**Published:** 2025-06-13

**Authors:** T. Nkomo, Z. Udwadia, D. Vambe, A. van Rie, S.S. Thi, J. Stillo, A. Stambekova, A. Sinha, M.L. Rich, A. Reuter, J. Patel, R. Otto-Knapp, I. Motta, A. Mesic, L. McKenna, S. Maru, E. Lessem, C. Lange, N. Kiria, Y. Kherabi, G. Günther, L. Guglielmetti, T. Decroo, L. Chen, A. Ashesh, A. Abubakirov, J. Furin

**Affiliations:** ^1^ National Tuberculosis Control Program, Manzini, Eswatini;; ^2^ Hinduja Hospital and Research Center, Mumbai, India;; ^3^ Baylor College of Medicine, Mbabane, Eswatini;; ^4^ University of Antwerp, Antwerp, Belgium and Stellenbosch University, Stellenbosch, South Africa;; ^5^ USAID, Mbabane, Eswatini;; ^6^ Wayne State University, Detroit, MI, USA;; ^7^ Partners In Health, (Almaty, Kazakhstan and Boston, MA, USA);; ^8^ Médecins Sans Frontières;; ^9^ German Central Committee to Combat Tuberculosis, Berlin, Germany;; ^10^ MRC Clinical Trials Unit, University College London, London, UK;; ^11^ Institute for Tropical Medicine, Antwerp, Belgium;; ^12^ Treatment Action Group, New York, NY, USA;; ^13^ New York City Department of Health, New York, NY, USA;; ^14^ Global Tuberculosis Community Advisory Board, NY, USA;; ^15^ Center for Tuberculosis Research, Department of Medicine, Johns Hopkins University, Baltimore, MD, USA;.; ^16^ Clinical Infectious Diseases, Research Center Borstel, Leibniz Lung Center, Borstel, Germany;; ^17^ Clinical Tuberculosis Unit, German Center for Infection Research (DZIF), Hamburg-Lübeck-Borstel-Riems, Germany;; ^18^ National Center for Tuberculosis and Lung Disease, Tbilisi, Georgia;; ^19^ Infectious and Tropical Diseases Department, Bichat-Claude Bernard Hospital, Assistance Publique-Hôpitaux de Paris, Université Paris Cité, Paris, France;; ^20^ Université Paris Cité, Inserm, IAME, Paris, France;; ^21^ Department of Pulmonology, Allergology and Clinical Immunology, Inselspital, Bern University Hospital, Bern, Switzerland;; ^22^ Department of Medical Sciences, Faculty of Health Sciences, University of Namibia, Windhoek, Namibia;; ^23^ Sorbonne Université, National Institute of Health and Medical Research (INSERM), U1135, Centre d’Immunologie Et Des Maladies Infectieuses, Paris;; ^24^ University of California, San Francisco, San Francisco, CA, USA;; ^25^ Survivors Against TB, New Delhi, India;; ^26^ Harvard Medical School, Boston, MA, USA.

**Keywords:** tuberculosis, resistance, treatment regimen, holistic support

## Abstract

**BACKGROUND:**

Strains of *Mycobacterium tuberculosis* with resistance to the new and repurposed drugs included in the all-oral shorter TB regimens recommended by WHO for the treatment of multidrug-resistant/rifampicin-resistant TB (MDR/RR-TB) are becoming increasingly common globally. When strains of *M. tuberculosis* have resistance to one or more of these drugs (bedaquiline, linezolid, third-generation fluoroquinolones, delamanid, pretomanid, or clofazimine), they are more challenging to treat.

**METHODS:**

In the absence of trial data on how to care for these individuals, a group of clinical, programmatic and civil society experts came together to generate a series of best clinical practices. These practices are based on the published literature and on experience caring for individuals with these forms of TB.

**RESULTS:**

We discuss best clinical practices in the following areas: 1) drug susceptibility testing; 2) regimen design; 3) adverse event monitoring and management; 4) special populations; 5) shared decision making and informed consent; 6) holistic packages of support; and 7) pre-approval access/compassionate use of newer TB compounds.

**CONCLUSION:**

While we await systematic studies of treatment approaches to generate the necessary evidence base, the clinical practices described here can be used to guide the programmatic care of people with strains of *M. tuberculosis* that have expanded resistance

Treatment for multidrug-resistant/rifampicin-resistant TB (MDR/RR-TB) has significantly improved over the past five years.^1^ There are now multiple all-oral options available to treat most people diagnosed with MDR/RR-TB in 6 to 9 months.^2^ Most of these regimens utilize combinations of bedaquiline (Bdq), linezolid (Lzd), a third-generation fluoroquinolone (either levofloxacin [Lfx] or moxifloxacin [Mfx]), a nitroimidazole (either pretomanid [Pa] or delamanid [Dlm]), and/or clofazimine (Cfz).^3^ Strains of *Mycobacterium tuberculosis* resistant to one or more of these medications^4–17^ present complications for treatment and care^18^ and increase the risk of TB related morbidity, mortality and treatment-related adverse events.^19–,22^ No guidelines outline the treatment of individuals with *M. tuberculosis* strains resistant to the newer and repurposed drugs, referred to here as ‘expanded resistance’. Care of these individuals is complicated in the absence of an evidence-based approach.^23–27^ To address this gap, a group of TB clinical experts, impacted communities, national TB program representatives and civil society organizations formed a community of practice called the BETTER (Building Experience Treating Tuberculosis with Expanded Resistance) Project. BETTER has elaborated best clinical practices to optimize care of people with these forms of TB as described below.

## METHODS

The clinical care practices discussed in this paper are based on experiences of the authors supporting more than 50 countries in all WHO regions since newer drugs became available. The authors met five times online and once in person to review these practices and come to consensus about their use. When there was not agreement, discussion was had amongst all authors until there was agreement. In addition to developing these series of care practices in discussion with one another, the authors also discussed practices with TB programs, consulted existing literature, and listened to the experiences of people and communities affected by these forms of TB. The authors searched the peer-reviewed literature using OVID, MedLINE and PubMED databases from January 1, 2016 to January 31, 2025, using the terms: ‘rifampicin-resistant tuberculosis’ or ‘drug-resistant tuberculosis’ with ‘bedaquiline’, ‘delamanid’, ‘pretomanid’, ‘clofazimine’, ‘linezolid’ and ‘resistant’. There are some practices shared here for which there is limited implementation experience. Such practices are included because they may be necessary for people with limited treatment options.

The term ‘best practices’ is used here because, for each of the principles discussed, one or more of the authors has used the approach in treating someone with TB that has expanded resistance. We did not use the term ‘recommendations’ to differentiate this work from the more formal guideline development/guideline review processes undertaken to generate recommendations. Expanded resistance was defined as MDR/RR-TB with additional phenotypic or genotypic resistance to one or more of the following; Bdq, Lzd, Cfz, Dlm, and/or Pa. Our paper includes some strategies for people with TB that has fluoroquinolone resistance, but since the WHO defines these as ‘pre-extensively drug-resistant TB’ and offers specific guidance on how to treat such types of TB, we focus on expanded resistance.

## 1. DRUG SUSCEPTIBILITY TESTING

Drug susceptibility testing (DST) to the drugs in the WHO-recommended, all-oral, shorter regimens (Bdq, Lzd, Lfx, Mfx, Cfz, Pa, and Dlm) should ideally be made available to all persons with MDR/RR-TB being offered these regimens. Phenotypic testing for many of these compounds is available^28^. Alternatively, targeted next-generation sequencing (tNGS) or whole genome sequencing (WGS) can identify strains of *M. tuberculosis* with expanded resistance.^29^ Of note, cross resistance is common between Bdq and Cfz and between the nitroimidazoles Pa and Dlm. There is a need for rapid expansion of access and interpretation of these methods.^30^ While building capacity, the following individuals should be prioritized for expanded DST:^31^

1. People with a history of treatment with any of these drugs, particularly those who experienced unsuccessful treatment outcomes or who had treatment interruption, especially if treatment interruption occurred early during treatment or when the culture was still *M. tuberculosis p*ositive;
2. People on treatment with a regimen that includes these drugs who have suboptimal bacteriologic or clinical response;
3. People diagnosed with MDR/RR-TB who are in close contact with individuals with confirmed resistance to these drugs or any of the risk factors described above.

## 2. REGIMEN DESIGN

Most people diagnosed with strains of *M. tuberculosis* with expanded resistance will need an individualized treatment regimen. Whenever possible, this should be based on the observed DST profile. Individuals eligible for individualized treatment regimens include:

1. People with MDR/RR-TB that have confirmed resistance to Bdq, Lzd, Pa, Dlm and/or Cfz with or without resistance to Lfx and/or Mfx. This could be from a DST done at baseline or during the course of treatment if the person is having a sub-optimal clinical or bacteriologic response to treatment.
2. People with possible resistance to Bdq, Lzd, Pa, Dlm and/or Cfz with or without resistance to Lfx and/or Mfx. This would include people who were treated with these drugs previously and did not have a successful treatment outcome. It would also include people who are on a regimen containing these drugs who are experiencing sub-optimal clinical or bacteriologic response to treatment.
3. Close contacts of people with confirmed or possible resistance to Bdq, Lzd, Pa, Dlm and/or Cfz with or without resistance to Lfx and/or Mfx.
4. People who develop toxicity leading to permanent discontinuation of one or more of the drugs in the WHO-recommended, all-oral, shorter regimen they are receiving.

An empiric individualized regimen should be administered while awaiting the DST results. Empiric treatment regimens can be adjusted once the DST results are available. If access to DST is not possible, the empiric regimen can be continued until the end of treatment. Following WHO recommendations, treatment duration for individualized regimens will usually be 15–17 months after culture conversion, depending on the resistance pattern, treatment response, the extent of disease and co-morbidity considerations. Individualised regimen design must consider several criteria. These include: 1) the 2018 WHO grouping of drugs;^32^ 2) drug penetration into different tissues and lesions;^33^ 3) the potential bactericidal and sterilizing activity of the remaining drugs;^34^ 4) the known or presumed resistance pattern; 5) the extent of disease;^35^ 6) any known co-morbidities;^36^ and 7) the toxicity profiles/potential drug-drug interactions of proposed treatment.^37^

Bactericidal drugs play a critical role in the early phase of treatment, while sterilizing drugs are needed to achieve a durable, relapse-free cure.^38^ Some drugs may not be as effective at killing mycobacteria but are able to stop their growth, and these agents are known as ‘bacteriostatic’ drugs. When the number of bactericidal agents in the regimen is low, drugs with primarily bacteriostatic activity can help prevent the emergence of resistance to the bactericidal agents in the regimen. While these concepts have historically been important in developing regimens for treating TB, the reality of determining whether an individual drug is bactericidal, sterilizing, or both requires extrapolating from various types of data, much of which come from studies of combination regimens. Although this is a simplification of a nuanced topic, Table 1 summarizes the primary roles of different drugs.

**Table 1.** Bactericidal and sterilizing activity of second-line TB drugs.

Category	Drugs
Drugs with both bactericidal and sterilizing activity	Bdq, Lfx, Lzd, Mfx, Pa* (or Dlm*)
Primarily bactericidal drugs	Am, Carbapenems + clavulanic acid, Eto**, high-dose Inh (if no *katG* mutation)**
Primarily sterilizing drug	Pza, Cfz
Primarily bacteriostatic drugs with limited/no sterilizing activity	Cs, Emb, PAS

* The sterilizing activity of these drugs is still being investigated.

** These drugs are generally considered less potent bactericidal agents compared to others in the same category.

Am = amikacin; Bdq = bedaquiline; Cfz = clofazimine; Cs = cycloserine; Dlm = delamanid; Emb = ethambutol; Eto = ethionamide; Inh = isoniazid; Lfx = levofloxacin; Lzd = linezolid; Mfx = moxifloxacin; Pa = pretomanid; PAS = para-aminosalicylic acid; Pza = pyrazinamide.

In addition to choosing drugs for a regimen based on drug attributes and clinical patient characteristics considerations, clinical best practice should also take the preferences of the person being treated into account. This is particularly important when hospitalization is necessary for drug delivery or if the drug is associated with a risk of permanent disability.^39^ Additional considerations in regimen design include drug availability, registration status of a drug, storage requirements and other administration factors. The number of drugs to be included in an individualized regimen will vary depending on the extent of disease and co-morbidities. Of note, not all the drugs included in the initial regimen will be given for the entire length of treatment (i.e., the carbapenems/clavulanic acid or amikacin [Am] may be given for shorter durations). People with non-severe disease (isolated to the cervical or intra-thoracic lymph nodes without airway compression or that involves only unilateral pulmonary infiltrates in less than one lobe without any cavities or miliary disease)^40^ may have a lower bacillary burden and receive a regimen of four likely effective drugs whereas those with more severe disease (bilateral or cavitary pulmonary TB, severe extrapulmonary TB such as TB meningitis, abdominal or disseminated TB) should receive at least five likely effective drugs. In the absence of DST results, a ‘likely effective’ drug is one that has not been used by the individual in the past, has no cross resistance with drugs used in the past, and has not been used in a close contact with a strain of *M. tuberculosis* that has resistance to the drug.^41^ The actual number of drugs people receive, however, could be higher, as some drugs may be included in the regimen even if they are not ‘likely effective’. For example, Bdq might be given to a patient while awaiting DST results even though the patient has received Bdq in the past but an additional four or five drugs will be needed depending on disease severity.

We suggest a stepwise approach to designing an individualized regimen as there is no one-size-fits-all approach. The steps should be followed until the desired number of likely effective drugs is reached while maximizing agents with bactericidal and sterilizing properties. The approach builds on the principles described by WHO.^42^ It also suggests prioritizing certain group C drugs and considers some drugs that were not grouped. The steps below are also summarized in Table 2.

*Step 1: Include as many ‘core drugs’ as possible (Bdq, Lzd, 3*^*rd*^ *generation fluoroquinolones)*

Core drugs are Group A drugs that are both sterilizing and bactericidal and include Bdq, Lzd and the third-generation fluoroquinolones. These drugs should be included if susceptibility is documented or uncertain, but they should not be counted if they are not ‘likely effective’ drugs. Whether or not these drugs should be included even if they are not counted as effective should also be based on the pill burden, toxicity, and the preferences of people undergoing treatment.

*Step 2: Add oral agents with bactericidal or proven bacteriostatic activity (Pa, Dlm, cycloserine [Cs], high-dose isoniazid [Inh], ethionamide[Eto])*

These drugs include nitroimidazoles (Pa or Dlm) and/or Cs. Cs is prioritized here as a WHO Group B drug. Many providers regard Pa as a potent anti-TB drug, and its lower cost compared to Dlm is appealing. However, Pa has only been tested in combination with other agents, has not been categorized in a WHO group, and when designing an individualized regimen, Dlm may be preferred. Dlm is a WHO Group C nitroimidazole agent, and unlike Pa. can be given to children/adolescents and during pregnancy. Depending on the resistance profile, add either high-dose Inh (if no *katG* mutation) or ethionamide (Eto) (if only a *katG* mutation).

**Table 2.** Stepwise approach to individualized regimen design.

Step	Drug	WHO Group (if applicable)	Comment
**1: Include as many core drugs as possible**			
	Bedaquiline	A	May include even if not a ‘likely effective’ drug but should then not be counted in drug total.
	Linezolid	A	May include even if not a ‘likely effective’ drug but should then not be counted in drug total. Do not plan systematic dose reduction if tolerated.
	Third-generation fluoroquinolones (Lfx or Mfx)	A	May include even if not a ‘likely effective’ drug but should then not be counted in drug total.
**Step 2: Add oral agents with bactericidal or proven bacteriostatic activity.**			
	Cycloserine	B	Phenotypic and genotypic testing for Cs may be challenging to do and interpret.
	Pretomanid or Delamanid	Pa not grouped as only given in combination with other agents; Dlm is group C	Pa and Dlm are both nitroimidazoles, and only one should be used. Pa should not be used with Pza, as there is an increased risk of hepatotoxicity. Dlm is the nitroimidazole of choice in children, adolescents, and during pregnancy.
	High-dose isoniazid	Not grouped	Should only be used in the absence of *katG* mutation.
	Ethionamide	C	Should only be used in the absence of an *inhA* mutation or other mutations associated with phenotypic ethionamide resistance.
**Step 3: Add oral agents with sterilizing or potential sterilizing activity**			
	Clofazimine	B	Cross resistance between Bdq and Cfz is common, and therefore Cfz should not be used in settings of Bdq resistance.
	Pyrazinamide	C	Might have a synergistic effect with Bdq. Should not be used with Pa because of increased risk of hepatotoxicity. Use in the setting of documented resistance is controversial.
**Step 4: Add one or two injectable agents for their bactericidal activity**			
**Step 4a: Add amikacin (Am)**	Amikacin	C	Use three times per week at dose 25mg/kg to decrease toxicity^68^, which is associated with the cumulative dose^1^. Can be administered intramuscularly. Consider adding lidocaine to the injection if given intramuscularly. Administration in slow (60min) infusion with 5% glucose could also be considered (without lidocaine). Perform audiometry at least once a month (ideally, twice per month). Avoid use in children and in pregnancy, unless there are no other options.
**Step 4b: Add a carbapenem given in combination with clavulanic acid**	Carbapenem in combination with clavulanic acid	C	Some providers/programs prefer a carbapenem in combination with clavulanic acid over Am. Meropenem is preferred over imipenem. Ertapenem has less data to support its use in TB, but could be considered for intramuscular administration if long-term intravenous access is not possible. Carbapenems must always be given in combination with oral amoxicillin-clavulanic acid (usually administered 30 minutes prior to the carbapenem)
**Step 5 Consider other ‘back-up’ drugs when regimen options are extremely limited**			
	Ethambutol	C	Some providers may prioritize this drug over Step 4 agents if resistance is unlikely (e.g., no known Emb mutations), particularly when injectables are contraindicated or risk of toxicity is high. Prior use in the patient is not a reliable indicator of effectiveness due to widespread background resistance.
	Para-aminosalicylic acid	C	PAS is a toxic drug that is not considered to be very potent by many providers.
**Step 6: Consider pre-approval access/ compassionate use drugs**	Quabodepistat, ganfeborole, telacebec, BTZ-043, TBAJ-076, TBAJ-587		Some providers would use these drugs over the drugs in step 5, depending on access, patient preference, and tolerance.

Am = amikacin; Bdq = bedaquiline; Cfz = clofazimine; Cs = cycloserine; Dlm = delamanid; Emb = ethambutol; Eto = ethionamide; Inh = isoniazid; Lfx = levofloxacin; Lzd = linezolid; Mfx = moxifloxacin; Pa = pretomanid; PAS = para-aminosalicylic acid; Pza = pyrazinamide; QTcF = QT interval with Fridericia correction.

*Step 3: Add oral agents with sterilizing or potential sterilizing activity. (Pza, Cfz)*

Pza should not be used with Pa because of an elevated risk of hepatotoxicity. If Pza is used, Dlm is the preferred nitroimidazole. If there is known or possible resistance to Bdq, then Cfz resistance is likely and Cfz is not likely to be effective.

*Step 4: Add a bactericidal injectable agent*

*Step 4a: Add amikacin (Am)*

If resistance is unlikely and if monitoring for hearing loss is available. Expanded resistance may warrant Am use even though this agent is difficult to administer using modalities acceptable to people with TB. The duration of Am will depend on toxicity, response to therapy, and the availability of other drugs in the regimen. Unless specific contraindications exist, the minimum duration is considered to be four months. Some programs may prefer to prioritize a carbapenem with clavulanic acid (see step 4b), depending on logistics and patient preferences.

*Step 4b: Add a carbapenem given in combination with clavulanic acid*

Carbapenems should be given in combination with clavulanic acid, usually administered as oral amoxicillin-clavulanic acid 30 minutes prior to the intravenous carbapenem.^43^ Although these agents are less desirable as they must be given intravenously (via a port-a-cath or peripherally inserted central catheter system) more than once a day, expanded resistance may warrant their use. Meropenem is preferred over imipenem^44^ if there is a choice of agents. Duration of use has not been well-established. A minimum duration is usually eight weeks but if there are limited treatment options, six to 12 months may be required.

*Step 5: Consider other ‘back-up’ drugs when regimen options are extremely limited*

Usually, drugs added in this step have primary bacteriostatic activity and an unclear or no sterilizing role, and are used as ‘back-up’ in case one of the drugs selected in the previous steps is less active than presumed. Examples of such drugs are para-aminosalicylic acide (PAS) and ethambutol (Emb), if susceptible. Emb may cause optic nerve damage and may be of concern if included in a regimen with LZD which has similar toxicity while PAS has been associated with gastrointestinal (GI) side effects. Emb often has a high background of resistance and does not have reliable DST methods which limits the ability to count it as an effective drug in the regimen. These agents should only be considered when there are no other options. However, they may be preferred to the injectable agents in step 4 if there is toxicity or difficulty administering those drugs.

*Step 6: Consider pre-approval access/compassionate use drugs*

Agents that have already completed phase 2a studies include quabodepistat,^45^ ganfeborole,^46^ BTZ-043,^47^ and telacebec.^48^ Other promising agents that should be prioritized for pre-approval access/compassionate use programs include TBAJ-876 and TBAJ-587.^49^ Additional discussion on step 6 is presented in the section below on pre-approval access to novel compounds.

### Higher dosing of second-line drugs

In most patients, it is likely that a regimen consisting of four or five likely effective drugs can be constructed. Higher dosing of some drugs could be considered in those patients with very limited options, although data on the use of higher drug doses is absent for most of the second-line drugs. This is an area with limited implementation experience, but it is an approach being used by some providers when there are no other treatment options. The rationale for higher doses of drugs includes: 1) there may be mutations that only confer resistance to lower drug concentrations; 2) higher drug concentrations could improve penetration into fibrotic/cavitary lesions; and 3) higher drug concentrations could maximize the time the drug levels are above the dose needed to kill *M. tuberculosis*.

If higher doses of any second-line drugs are given, more frequent monitoring is essential, with ongoing revisitation of the risks and benefits and reporting of adverse events. Table 3 summarizes some of the higher dose options of the second-line TB drugs.

**Table 3.** Higher dose options of second-line drugs.

Drug	High-dose option	Monitoring on high-dose option	Comment
Bedaquiline	500mg loading dose for 14 days followed by 200 mg daily	More frequent monitoring of QTcF interval (i.e. every 14 days)	Microbiologic or genomic data may not reliably indicate when higher doses of Bdq are effective. If there is detection of an *atpE* mutation, then do not use Bdq at any dose.
Clofazimine	300mg daily	More frequent QTcF monitoring, especially if used in combination with other QTcF prolonging drugs	
Isoniazid	1015mg/kg/day or 15–20mg/kg/day if used in combination with Cs and there is low risk for neurotoxicity, as Cs may lower Inh concentrations.	Monthly monitoring for peripheral neuropathy	Should give with vitamin B6 (25–75mg daily) to prevent peripheral neuropathy. Do not use if a *katG* mutation is detected. Of note, some *inhA* mutations may stil confer resistance to Inh at higher doses.
Levofloxacin	30mg/kg/day	More frequent QTcF monitoring, especially if used in combination with other QTcF prolonging drugs	
Linezolid	1200mg daily	Complete blood count, visual acuity/color vision screening, and screening for peripheral neuropathy every 14 days	The toxicity of this dose of Lzd has been well established. High dose should only be used if there are no other options.
Moxifloxacin	12–15mg/kg/day	More frequent QTcF monitoring, especially if used in combination with other QTcF prolonging drugs	

Am = amikacin; Bdq = bedaquiline; Cfz = clofazimine; Cs = cycloserine; Dlm = delamanid; Emb = ethambutol; Eto = ethionamide; Inh = isoniazid; Lfx = levofloxacin; Lzd = linezolid; Mfx = moxifloxacin; Pa = pretomanid; PAS = para-aminosalicylic acid; Pza = pyrazinamide; QTcF = QT interval with Fridericia correction.

### Areas for future research

*Ancillary medications*: there are theoretical and experimental data suggesting that the efflux pump by which resistance to Bdq and Cfz is mediated can be overcome using calcium channel blockers such as verapamil.^50^ This is a potentially important area for future research. No established verapamil dose has been defined, and it is an area of limited implementation experience.

*Mutations of uncertain significance;* If mutations classified as ‘of uncertain clinical significance’ in the WHO catalogue of mutations in *M. tuberculosis*^51^ are detected, a phenotypic DST should be requested. Consultation with an expert familiar with the interpretation of mutations should be sought. Some of the core/group A drugs may be given in settings of uncertainty on the clinical significance of a mutation in a candidate resistance gene, as the benefit of retaining the drug may outweigh the risk.^52^ If used, the drug should not be counted as a likely effective drug, and the regimen should then contain more than four or five drugs proven to be effective. When currently approved tNGS assays are used, information on nitroimidazoles will be lacking. Whether or not these drugs should be included should also be based on prior exposure, pill burden, toxicity and the preferences of people undergoing treatment.

## 3. ADVERSE EVENT MONITORING AND MANAGEMENT

Many people with forms of TB that have expanded resistance will require treatment with drugs that are associated with high rates of toxicity.^53^ They may also need to receive higher doses of some drugs or require multiple drugs that have overlapping toxicity (such as QTcF prolongation), which may increase toxicity risk. For this reason, monitoring and management of adverse events must be included as part of the package of care offered to these individuals. Adverse events should also be reported to the relevant pharmacovigilance authorities as part of efforts to strengthen active drug safety monitoring and management.^54^

## 4. SPECIAL POPULATIONS

Most of the anti-TB drugs described above have been used in pediatric^55^ and pregnant and lactating populations.^56^ Howeve, Pa has not been given to children under the age of 14 years or to pregnant women. Special care should be taken when considering Am in these groups, given its harmful effects on the fetal ear and the consequences of hearing loss in children and adolescents. Except for Pa and Am, the best practices discussed in this document can be applied to children of all ages and in pregnancy and lactation. Ideally, these populations should receive Dlm as the nitroimidazole of choice if one is to be used in their treatment regimen. Pediatric and pregnant populations should also be considered for pre-approval access/compassionate use of new drugs, with ongoing discussions about the risks and benefits of various treatment approaches.

For children, it is important to use weight-based dosing of all the medications, which are specified in the WHO guidelines^57^ and in a ‘Field Guide’ developed by the Sentinel Project on Pediatric Drug-Resistant TB.^58^ Studies have also shown that children with non-severe forms of drug susceptible TB (i.e. TB that is isolated to the cervical or intra-thoracic lymph nodes without airway compression or that involves only unilateral pulmonary infiltrates in less than one lobe without any cavities or miliary disease) may need less intensive treatment in terms of composition and/or duration. Many children will not have bacteriologic confirmation of TB with expanded resistance, but they will be close contacts of people who have these forms of TB. In such situations, an individualized regimen should be constructed based on the DST results of the person with confirmed resistance with whom the child has been in contact. All efforts should be made to obtain samples from the child for bacteriologic confirmation, as DST patterns are not always 100% identical among close contacts. Child-friendly formulations should be used in young children whenever possible, and such formulations exist for almost all the second-line drugs (except Pa).

## 5. SHARED DECISION MAKING AND INFORMED CONSENT

Shared decision-making should be a routine part of TB service delivery. It is especially important in settings of expanded resistance since there is not yet a strong evidence-base to support any regimen or approach. Shared decision-making means eliciting and considering the needs, preferences, and values of the person seeking care so that these issues can be considered alongside the recommendations of the providers when designing a treatment regimen. It conceptualizes people living with TB and TB service providers as active, equal partners in all treatment decisions.^59^ How this is best accomplished will look different across various cultural contexts, and it will be important to adapt the general principles of shared decision making into locally suitable models of care. The first step in shared decision making is for the person undergoing treatment to be informed about their options. This process of informed consent is more expansive and ongoing than that typically used in TB care and cannot be a once-off event in which a form is signed for legal reasons. Rather, it is a continuous discussion grounded in honesty between providers and a person undergoing treatment. For people with TB that have expanded resistance and who have very limited treatment options, palliative end-of-life care should also be discussed.

## 6. HOLISTIC PACKAGES OF SUPPORT

Receiving a diagnosis of TB can be devastating and receiving a diagnosis of TB with expanded resistance, which is more difficult to treat, can be overwhelming. Many people facing TB with expanded resistance also face multiple social, economic, and psychological challenges that make the treatment journey a challenge.^60^ Acknowledging and working to address these issues in compassionate, equitable and comprehensive ways is as important as selecting the right drugs for the treatment regimens. This type of holistic care should not be seen as an optional addition to treatment of TB with expanded resistance but rather as a core part of service provision. Providing such care will mean that TB programs need to collaborate with other arms of government services (e.g., social protection, housing, integrated mental health support^61^) as well as non-governmental organizations. Professionalized and paid peer educators/supporters may be a highly effective way to provide many of these support services.^62^

## 7. PRE-APPROVAL ACCESS TO NOVEL COMPOUNDS

Some persons affected by TB with expanded resistance may have extremely limited options and may benefit from access to newer agents to enable effective individualized treatment.^63^ This can be done via compassionate use (CU) or pre-approval/expanded access (EA) programs.^64^ Companies, academic innovators and drug developers should design these programs in consultation with external TB experts who have relevant experience and impacted community groups. The goal of this collaborative process is to make the drugs available through CU/EA programs after phase 2b data shows promise of safety and efficacy. For example, the drug quabodepistat, which was shown to be active against TB and had an acceptable safety profile in phase 2b testing,^65^ should be ready to move into CU/EA use no later than the second half of 2025. TB programs will also need to make sure they proactively explore, along with relevant regulatory authorities, developing or strengthening CU/EA programs in their settings. These programs should be free of costs to programs and people with TB, universally available, transparent, and flexible enough to integrate into a variety of regulatory environments, including ones that allow for expanded access on an individual named-patient basis and those that require access under a research protocol. Programs must allow for both single and concurrent use of novel compounds as clinically warranted. Finally, these programs should be funded as part of the development of new drugs by drug companies and donors, rather than developed hastily after-the-fact.

## CONCLUSION

Although most people with MDR/RR-TB can now be treated with safe and effective WHO recommended, all-oral shorter treatment, a growing number have strains of *M. tuberculosis* resistant to one or more of the drugs in these regimens. These individuals require compassionate, comprehensive care guided by the best available – even if limited – evidence. The authors came together to elaborate such best clinical practices, while recognizing the limitations of our work, including lack of a formal evidence base and limited implementation experience. There is an urgent need for operational and clinical trial research to better address expanded resistance, including issues people face along the TB diagnostic and care cascade and their risk of post-TB lung disease.^66, 67^ While awaiting the results of such studies, the framework presented here offers a practical approach to caring for individuals with expanded resistance. We hope this guidance supports clinicians and people with these types of TB in making informed treatment decisions, while acknowledging that care must be tailored to local and regional contexts.
